# Antibiotics’ Use in Thailand: Community Pharmacists’ Knowledge, Attitudes and Practices

**DOI:** 10.3390/antibiotics10020137

**Published:** 2021-01-31

**Authors:** Budh Siltrakool, Ilhem Berrou, David Griffiths, Saleh Alghamdi

**Affiliations:** 1 Department of Clinical, Pharmaceutical and Biological Sciences, School of Life and Medical Sciences, University of Hertfordshire, Hatfield AL10 9AB, UK; puttapong@go.buu.ac.th (B.S.); d.g.griffiths@herts.ac.uk (D.G.); 2 Faculty of Health & Applied Sciences, University of the West of England, Bristol BS16 1DD, UK; 3 Department of Clinical Pharmacy, Faculty of Clinical Pharmacy, Albaha University, Albaha, Saudi Arabia; saleh.alghamdi@bu.edu.sa

**Keywords:** community pharmacy, antimicrobial resistance, Thailand

## Abstract

Thailand spends $203 million on antibiotics every year, and patients can still access antimicrobials over the counter without a prescription. Community pharmacy plays a pivotal role in improving access and ensuring the appropriate use of antimicrobials. However, little is known about current practices in this setting. This study aims to assess Thai community pharmacists’ knowledge, attitudes and practices (KAP) regarding antimicrobials’ use and resistance. A cross-sectional study was conducted in Bangkok and Chonburi province in 2017 using an online self-administered questionnaire. The questionnaire was completed by 372 community pharmacists (71.4% response rate). The most commonly encountered infections in the community were upper and lower respiratory tract infections. The most commonly dispensed antimicrobials were broad-spectrum antibiotics including aminopenicillins and fluoroquinolones. Thai pharmacists have a good knowledge, attitude, and practice regarding antimicrobials’ use and resistance. They dispense anti-microbials in line with local guidelines, although international guidelines may not indicate anti-biotics for viral self-limiting infections. While community pharmacy in Thailand could be the most accessible healthcare resource for patients, inappropriate provision of antimicrobials for self-limiting viral infections by pharmacists will increase antimicrobial resistance. This highlights the need for updated guidance and improved pharmacists’ training.

## 1. Introduction

Global consumption of antibiotics increased by 65% from 2000–2015, fueling a growing epidemic of antimicrobial resistance and the emergence of novel multi-drug-resistant strains, particularly in low- and middle-income countries (LMIC) [1]. It is estimated that antimicrobial resistance infections are a cause of death to around 700,000 people a year worldwide; a toll that would rise to 10 million people by 2050, with a huge economic burden of USD 60 to100 trillion [2]. In Thailand, antimicrobial resistance infections cause 3.24 million additional hospitalisation days and 38,481 fatalities annually. Thailand spends USD 84.6–202.8 million a year on antibiotics and has a productivity loss as a result of morbidity and mortality of at least USD 1.3 billion [3,4].

Thailand has a population of around 68 million people [5]. The Thai National Statistics Office (NSO) reports that households spent 0.9% of their expenses on purchasing self-medication including antimicrobials in 2013, and this increased to 1.4% in 2016 [5]. Thai patients can obtain antimicrobials without a prescription from community pharmacy, and so far, legislations allow community pharmacists to dispense antimicrobials without a prescription [6,7]. The Community Pharmacists Association (CPA) and National Health Security Office (NHSO) encourage the involvement of community pharmacy in health promotion and disease prevention projects [8,9]. However, the provision of vaccination service (such as flu vaccination) remains prohibited in Thai pharmacies [7].

Given that antimicrobial resistance is largely driven by the inappropriate use of antimicrobials, pharmacists are uniquely positioned to improve the utilisation of antimicrobials and preserve their efficacy. Community pharmacists in particular are key drivers to improving antimicrobial stewardship in contexts like Thailand, where pharmacies are the most cost-effective, accessible and sometimes the only healthcare provider available [10,11,12,13]. Evidence support the impact of pharmacists’ interventions on reducing antimicrobial resistance [12,14,15,16]. However, evidence also indicate that in countries and settings where antimicrobial resistance rates are soaring, pharmacists may lack awareness and can have inadequate knowledge of antimicrobial resistance. For example, community pharmacists do not frequently consider that infections encountered in the community often have viral causes, and dispensing antibiotics to treat them will further increase antimicrobial resistance. This has been reported in Thailand [17,18] and other countries in the region [19].

The Thai government devised a number of measures to tackle the issue of antimicrobial resistance. These include a national strategy to combat antimicrobial resistance using a one-health integrated approach in 2017 [20], the antibiotic smart use project [21], and ensuring that Thai pharmacists are competent to provide antimicrobials appropriately as part of their competency framework [22]. In addition, new pharmacy laws and regulations were introduced including the Good Pharmacy Practice in community pharmacy [23], the continuing pharmacy education (CPE) [24], and the 5-year National Strategic Plan on Antimicrobial Resistance in Thailand [13]. These changes are anticipated to affect the current and future of community pharmacy practice in antimicrobial provision. More importantly, pharmacy education in Thailand has also dramatically changed since 2009 [25]. From 2014, all new pharmacists have to graduate with a 6-year PharmD degree based on a new and enhanced 6-year curriculum, providing in-depth knowledge and a focus on improving pharmacists’ clinical skills. The newly qualified pharmacists are now expected to have more clinical skills and be able to deliver clinically focused services and antimicrobial stewardship in their practice [25]. This study aims to assess community pharmacists’ knowledge, attitudes, and practice (KAP) in relation to non-prescription antimicrobial use and antimicrobial resistance.

## 2. Results

A total of 521 community pharmacists in Bangkok and Chonburi province were invited to participate in the study, of whom 372 pharmacists completed the online questionnaire, between May and June 2017, providing a response rate of 71.4%. The average age of the participants was 32.02 (±5.81) years. The majority were female (*n* = 260, 69.9%), graduated with a Bachelor of pharmacy 5 year programme (*n* = 287, 77.2%), and worked in independent pharmacies (*n* = 233, 62.6%). Around 23% of the participants hold a PharmD certificate, following a 6 year programme. The demographic data of the participants are presented in Table 1.

### 2.1. Current Antimicrobials’ Dispensing Practice

**Figure 1:**
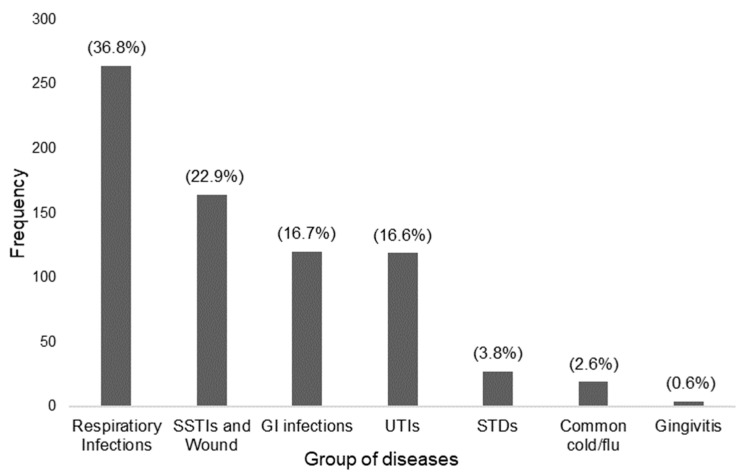
Commonly encountered infections in community pharmacies in Thailand. Respiratory infections included lower respiratory infections, upper respiratory infections, bronchitis, nasopharyngitis, otitis media, pharyngitis, rhinitis, sinusitis, sore throat, and tonsillitis. SSTIs (Skin and Soft Tissue Infections) and wounds included abscess, acne, bacterial skin infection, cellulitis, conjunctivitis, hordeolum, herpes simplex, impetigo, and tinea. UTIs (Urinary Tract Infections) included cystitis. GI (Gastro-Intestinal) infections included diarrhoea. STDs (Sexually Transmitted Diseases) included candidiasis, chlamydia, vaginitis, and gonorrhoea.

Community pharmacists were asked about the most commonly encountered infections in their practice setting for which they needed to dispense antimicrobials (without a prescription) using an open-ended question. A total of 717 responses were recorded. These were coded and grouped as shown in Figure 1.

**Figure 2:**
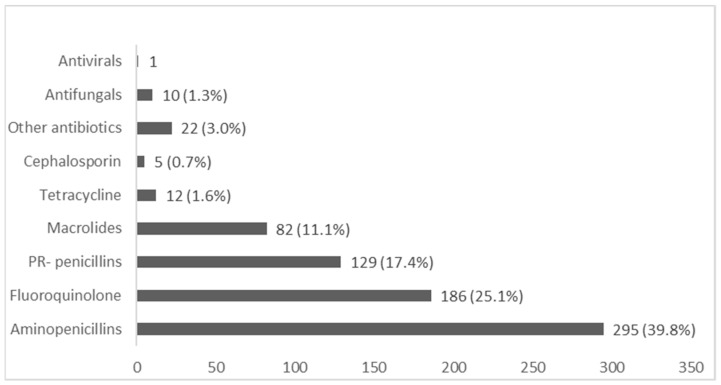
Commonly dispensed antimicrobials in community pharmacy. Aminopenicillin; Amoxicillin (*n* = 242), Amoxicillin/Clavulanic acid (*n* = 52), and Ampicillin (*n* = 1), Fluoroquinolone; Norfloxacin (*n* = 146), Ciprofloxacin (*n* = 28), Ofloxacin (*n* = 12), Penicillinase-resistant (PR)-penicillin; Dicloxacillin (*n* = 122), Cloxacillin (*n* = 7), Macrolide; Roxithromycin (*n* = 61), Azithromycin (*n* = 20), Erythromycin (*n* = 1), Tetracycline; Doxycycline (*n* = 9), Tetracycline (*n* = 3), Cephalosporin; Cephalexin (3), Cefuroxime (*n* = 1), Cephalosporin (*n* = 1), Other Antibiotics; Clindamycin (*n* = 11), Co-trimoxazole (*n* = 1), Metronidazole (*n* = 10), Antifungal; Clotrimazole (*n* = 4), Ketoconazole (*n* = 3), Fluconazole (*n* = 2), antifungal cream (*n* = 1), Antiviral; Acyclovir (*n* = 1).

Community pharmacists provided a total of 742 responses when asked about the most commonly dispensed antimicrobials in their practice setting. Amoxicillin, Amoxicillin/Clavulanic acid, and Ampicillin, grouped as aminopenicillins, were the most commonly dispensed antibiotics (39.8%), Followed by Fluoroquinolone (25.1%) and Penicillinase-resistance penicillin (17%). Findings are shown in Figure 2.

**Figure 3:**
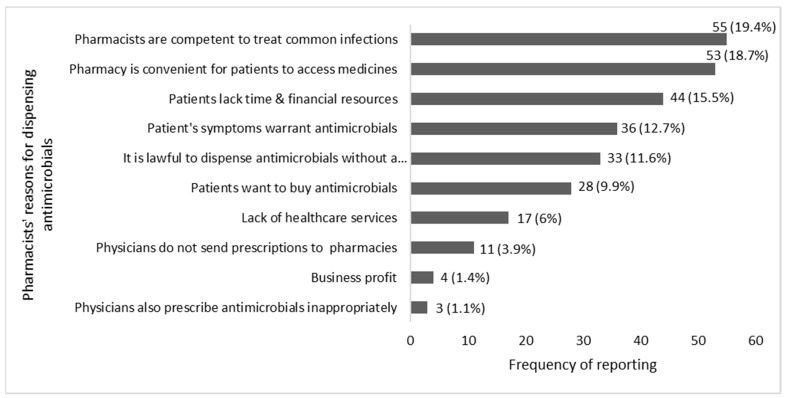
Reasons for dispensing antimicrobials without a prescription in community pharmacy in Thailand.

Pharmacy legislations in Thailand allow community pharmacists to dispense antimicrobials without a prescription. The participants were asked, using an open-ended question, to provide their reasons for dispensing antimicrobials without a prescription. The top five reasons were as follows: Pharmacists are competent to treat common infections in the community (19.4%), the pharmacy is the most convenient place for patients to access medicines (18.7%), patients lack time and financial resources to see a physician (15.5%), patients present symptoms that require antimicrobial treatment (12.7%), and it is lawful to provide antimicrobials without a prescription (11.6%). These and other reasons are shown in Figure 3.

### 2.2. Community Pharmacists’ Knowledge, Attitude and Practice in Antimicrobials’ Use and Resistance

#### 2.2.1. Knowledge of Antimicrobials’ Use and Resistance

Cronbach’s alpha was used to measure the internal consistency. The knowledge, attitudes, and practice sections scored 0.769, 0.783, and 0.742 respectively.

Knowledge of community pharmacists of antimicrobial resistance and use were evaluated in the knowledge section of the questionnaire. Table 2 shows the percentages of correct, incorrect, and uncertain answers for each item. Overall, the majority of participants provided correct answers to the first six statements. Question K1 had the lowest correct answers, as most participants were either incorrect or unsure of the term “superbugs”. Most participants would dispense antibiotics in the sore throat and wounds scenarios and would only dispense oral hydration therapy in the diarrhoea scenario. This is in line with “local guidance” but may contradict international guidelines.

#### 2.2.2. Attitudes Regarding Antimicrobials’ Use and Resistance

Community pharmacists generally agreed with positive attitude statements in relation to the appropriate use of antimicrobials and the issue of resistance. More than 90% of the participants selected “strongly agree” on the question of whether antimicrobial resistance is an important public health issue (A1) and whether patients who take antimicrobials need to be advised (A5). However, only less than half of the pharmacists agreed that dispensing antimicrobials without a prescription is a serious issue as shown in Table 3.

#### 2.2.3. Practices Relating to Antimicrobials’ Use and Resistance

Community pharmacists mostly demonstrate good practice in antimicrobials’ use amd resistance; however, they routinely dispense antimicrobials without a prescription. Table 4 shows the frequency of practices of participants in relation to reducing antimicrobial resistance and prudent use of antimicrobial agents. Community pharmacists are likely to educate their patients on antimicrobial resistance awareness (82.5%). However, only 32.2% of the pharmacists responded that they collaborate with other healthcare professionals for infection control and antimicrobial stewardship (P5).

#### 2.2.4. Relationship between Community Pharmacists’ Knowledge, Attitudes, and Practices

The knowledge, attitude, and practice (KAP) model suggests that each of the three dimensions affects and is affected by the others. Spearman’s rho correlation was used to explore the relationship between knowledge, attitude, and practice scores. No correlation was found between knowledge and attitude and knowledge and practice. A weak correlation was found between attitude and practice dimensions, with a score of 0.149 and *p*-value of 0.004 at 99% confident interval. Table 5 shows the results of correlation by Spearman’s rho test.

#### 2.2.5. Relationship between KAP Scores and Community Pharmacists’ Demographics

The knowledge, attitude, and practice scores were compared in relation to the participants’ demographics to explore the differences across gender, age, education, professional experience, type and locality of pharmacy. The findings are presented in Table 6. Overall, male pharmacists had better antimicrobial stewardship practices than female pharmacists. Pharmacists graduating with a PharmD degree had better knowledge, attitude and practice than pharmacists graduating with a BPharm. Pharmacists with varying lengths of professional experience had similar knowledge scores but varying attitudes and practices scores. Pharmacists who worked in chain pharmacies had better knowledge and attitude scores but scored similar practice scores to pharmacists working in independent pharmacies.

## 3. Discussion

Community pharmacists are expected to provide antimicrobials appropriately and take part in antimicrobial stewardship initiatives. This is the first study to assess community pharmacists’ knowledge, attitudes, and practices since the launch of the national strategy to combat antimicrobial resistance in 2017.

The most commonly dispensed antimicrobials in community pharmacy in Thailand are Aminopenicillins, Fluoroquinolone, and Penicillinase resistance penicillin. This is consistent with the information on the Center for Disease Dynamics, Economics and Policy (CDDEP) database and national surveillance data [26,27]. Respiratory tract infections were the most commonly encountered infections, which is consistent with previous research [10,12]. The most commonly dispensed antimicrobials are also often indicated for respiratory infections. However, cold and flu presentations to the community were minimal (2.6%). Cold and flu infections often present as respiratory infections for which community pharmacists are dispensing antibiotics despite the fact that these infections are often viral [12]. Allowing the provision of flu immunisation in community pharmacy can reduce the rates and spread of influenza infections. Educating patients and pharmacists about viral infections and how to manage symptoms without using antibiotics will improve and preserve the use of antibiotics.

Community pharmacists lack knowledge about “superbugs”, the prevalence of antimicrobial resistance and how it can be transferred. This highlights the urgent need to target education interventions at community pharmacists to improve awareness of the size and immediacy of the antimicrobial resistance threat and enhance efforts to curb it. Furthermore, in the knowledge section of the questionnaire, we provided pharmacists with three scenarios representing commonly encountered infections in the community. Although their responses corresponded to “good practice” and local guidelines, they do not necessarily indicate good antimicrobial stewardship. Guidelines should be updated to respond to local resistance rates, new evidence on treatment duration [28] and “tighter” criteria for antimicrobials dispensing, such as the fever pain score [29]. Other factors attributed to pharmacists’ provision of antimicrobials without a prescription should also be considered. First, the financial pressures community pharmacy businesses encounter [30,31]: community pharmacies tend to be business-oriented, and their business model is based on the sales of medicines and the need to satisfy customers in order to maintain competitiveness. The lack of financial means for their patients to seek medical advice for their condition from the physician [32,33] should also be considered when designing interventions to improve antimicrobial stewardship in community pharmacy. Of note, consumers’(mis)use of antimicrobials in the community increases antimicrobial resistance but can be extremely challenging to monitor and survey, especially in lower and middle-income countries that lack capacity and surveillance resources.

Overall, Thai community pharmacists have a good knowledge, attitude, and practice to combat antimicrobial resistance, and provision is at least in line with local guidance. These results concur with other studies in Thailand and Malaysia [10,34]. Pharmacists highlighted that they have access to Continuing Professional Development (CPD) activities in relation to antimicrobial stewardship, such as resources of the campaign to improve antibiotic use in community pharmacy, which was launched in 2012 [12]. However, if these campaigns are based on “out-of-date” guidelines and antimicrobials are still indicated for self-limiting infections [28,29], this is counterproductive in the fight against antimicrobial resistance.

Following the pharmacy education reforms and the increase in the number of pharmacists graduating with a PharmD [25], this study shows that these pharmacists have better knowledge, attitude and practices towards antimicrobials’ use and resistance. This is similar to findings by Donsamak [12], and in India by Ahmad and colleagues [35]. Pharmacists who worked in chain pharmacies had better knowledge and attitude scores than their colleagues who worked in independent pharmacies. This is not surprising given that chain stores often have the financial, governance, and staff training capacity that independent pharmacies may lack. However, no differences were observed in relation to practices. A study in India also found that chain pharmacies were less likely to dispense antimicrobials for diarrhoea in children compared to independent pharmacies [36]. While we found that the pharmacists’ age had no effect and the length of experience had a varying effect on pharmacists’ KAP, Donsamak [12] found that younger pharmacists and/or those with less experience were more likely to supply antibiotics appropriately. Furthermore, while we found that male community pharmacists demonstrate better antimicrobial stewardship practices, Donsamak’s study, involving a large proportion of female pharmacists, found gender to have no significant influence on the pharmacists’ wiliness to supply antibiotics (inappropriately).

The correlation results show that the knowledge and attitude scores of the pharmacists do not correlate, and that there is a weak relationship between attitude and practice scores. This is consistent with a previous study in pharmacy students that showed no and/ or a slight relationship between the three dimensions [35]. Other influencing factors outside the KAP model could affect pharmacists’ practice, including medication regulations, the health system, and patients’ pressure. Although the KAP model does not explain pharmacists’ behaviours, it can be a useful tool to measure pharmacists’ practice [14,37].

### Strengths and Limitations

This study improves our understanding of antimicrobials’ use and resistance in the community in Thailand. However, the KAP model does not investigate knowledge in anthropological terms such as culture-specific knowledge of illness or knowledge about the health system, e.g., quality and referral. In addition, the KAP model does not always explain why and how to practice [37]. Future research could examine the cause and effect of knowledge, attitude, and practice of the participants.

The questionnaire’s internal consistency and content validity were assessed by three Thai experts, so its content validity only extends to the Thai context. In the knowledge section, we used three clinical scenarios based on local guidelines. However, the clinician’s course of action in these scenarios will be different in another setting/country, so knowledge may be scored differently.

It is also important to consider the impact of social desirability bias on the pharmacists’ responses. The study findings demonstrate that pharmacists in Thailand have a good knowledge, attitudes, and practice in relation to antimicrobials’ use and resistance. However, this could be over-reporting of desirable answers and may not reflect true actions.

The study was conducted in two provinces in Thailand: Bangkok and Chonburi, which represent 30% of pharmacies in the country. The findings may not be generalisable, especially to rural parts of the country. The participants were recruited through online communities, email and social media. Some of the potential participants might not have been invited to participate if they could not/did not access online platforms.

## 4. Materials and Methods

### 4.1. Subjects and Setting

The study was conducted in two densely populated provinces of Thailand, Bangkok and Chonburi, which cover more than 30% of pharmacies in the country. Pharmacists who are part of the Thai Food & Drug Administration (FDA) and Community Pharmacy Association of Thailand (CPA) organisations were contacted by email to participate in the study. Pharmacists who were members of Chonburi Drugstore Network Group and the Community Pharmacy Service Group were invited to participate in the study through the social media platform LINE. The minimum sample size required was 358 participants, using the equation [38]:

*n* = (*Z*^2^ × *p*(1 − P))/e^2^/1 + (*Z*^2^ × P(1 − P))/e^2^ N (1)

where confidence interval is 95%, *Z* value is 1.96, proportion expected to respond is 50% or 0.5, margin error is 5% or 0.05, and population is 5082.

### 4.2. Questionnaire Development, Validation, and Piloting

An online self-administrated questionnaire was developed based on a thorough literature review of comparable studies to measure community pharmacists’ knowledge, attitudes and practice regarding antimicrobials’ use and resistance [24,25,34,39]. The questionnaire’s content validity was assessed by a panel of three experts in antimicrobial stewardship in community pharmacy and was piloted in a study involving 15 community pharmacists in Thailand. Cronbach’s alpha was used to measure the internal consistency; the knowledge, attitudes and practice sections scored 0.769, 0.783, and 0.742 respectively.

The 35-item questionnaire comprised of four main sections. The first covered sociodemographic data including personal background, education, professional experience, workplace location, and type of pharmacy. The second was an assessment of pharmacists’ knowledge of antimicrobial resistance and antimicrobial use, using 9 clinical scenarios from the antibiotic smart use guidelines in Thailand and previous studies in other healthcare professions [24,25,39]. Participants score 1 mark for a correct answer and 0 for an incorrect answer. The third component assessed the participants’ attitudes towards antimicrobials’ use and resistance, through community pharmacists’ agreement with 8 antimicrobials’ use and resistance statements using a 5-point Likert scale. These were adapted from a validated questionnaire by Roque et al. [40]. The last component examined the participants’ current actions of antimicrobials dispensing in pharmacy through 10 action statements and 3 open-ended questions. These statements were adapted from a study involving Malaysian community pharmacists [34]. Reverse coding was applied to negative statements.

### 4.3. Data Analysis

The Cronbach’s alpha and interclass correlation coefficients (ICC) were used as a test of reliability. Kolmogorov–Smirnov Test was used for normal distribution testing. Descriptive data were examined by the median, interquartile range (IQR), and Chi-squared test. Mann–Whitney U Test and Kruskal–Wallis test were used to describe associations between demographics, knowledge, attitude, and practice of the participants. Relationships between knowledge, attitude, and practice dimensions were analysed using Spearman’s correlation coefficient. Qualitative data were coded and presented as percentages.

## 5. Conclusions

Community pharmacists in Thailand have a good knowledge, attitudes, and practice in relation to antimicrobials’ use and resistance. However, the provision of antimicrobials in the community needs to be reviewed in light of updated guidance and antimicrobial stewardship plans. Pharmacists’ education reforms may improve pharmacists’ attitudes towards antimicrobials provision, but access to up-to-date resources and continuing professional education is necessary to improve antimicrobials’ use in the community.

## Figures and Tables

**Table 1:** Demographic data of the community pharmacists.

Demographic Data	
**Age (mean ± SD)**	32.02 ± 5.81
Less than 30 yrs, *n* (%) 30–40 yrs More than 40 yrs	145 (39.0) 185 (49.7) 42 (11.3)
**Experience (mean ± SD)**	5.46 ± 4.31
Less than 5 yrs, *n* (%) 5–10 yrs More than 10 yrs	180 (48.4) 152 (40.9) 40 (10.8)
**Pharmacy Degree***n* (%)	
BPharm/BSc in Pharm PharmD	287 (77.2) 85 (22.8)
**Postgraduate Degree***n* (%)	
None Master degree Doctoral degree	276 (74.2) 91 (24.5) 5 (1.3)
**Location***n* (%)	
Bangkok Chonburi	258 (69.4) 114 (30.6)
**Type of Pharmacy***n* (%)	
Independent Chain	233 (62.6) 139 (37.4)

**Table 2:** Frequency of correct, incorrect, and uncertain answers for statements about knowledge of antimicrobials’ use and resistance.

**Knowledge Items**	**Correct**		**Incorrect**		**Uncertain**	
	***n***	**%**	***n***	**%**	***n***	**%**
K1: “Superbugs” are microorganisms which generate antimicrobial resistance. They include bacteria, fungi, viruses or parasites.	176	47.3	98	26.3	98	26.3
K2: Resistance DNA in bacteria can be transferred to other bacteria by virus.	207	55.6	113	30.4	52	14.0
K3: Antimicrobial resistance in hospital setting is higher than community setting.	278	74.7	50	13.4	44	11.8
K4: The main objective of antimicrobial stewardship is to achieve the most effective clinical outcome with less toxicity and adverse reactions to antimicrobials.	283	76.1	80	21.5	9	2.4
K5: Penicillin, cephalosporin, and fluoroquinolone are β-lactam antibiotics. You need to consider Beta-lactamase producing bacteria.	262	70.4	107	28.8	3	0.8
K6: Patients who are allergic to Amoxicillin (Anaphylaxis type) should not use Cephalexin.	332	89.2	30	8.1	10	2.7
**Knowledge Items**	**Yes**		**No**		**Uncertain**	
	***n***	**%**	***n***	**%**	***n***	**%**
K7: Is it appropriate? When a pharmacist dispenses amoxicillin 1500 mg a day, 7 days for a 26-year-old male with allergic rhinitis, high-grade fever, rhinorrhoea, sore throat, and no known drug allergy.	297	79.8	61	16.4	14	3.8
K8: Is it appropriate? When a pharmacist dispenses only mineral powder for a 2-year-old boy with watery diarrhoea, no mucous/bloody stool, no fever, no vomiting, and no known drug allergy.	330	88.7	35	9.4	7	1.9
K9: Is it appropriate? When a pharmacist dispenses dicloxacillin 250 mg four time a day for 5 days to prevent an infection in case of a 24-year old male who has had a skin abrasion wound on his right arm without exudates for 2 days, limited to subcutaneous layer, mild tenderness, no swelling, no active bleeding, no fever, and no known drug allergy.	256	68.8	81	21.8	35	9.4

**Table 3:** Community pharmacists’ responses to attitude statements.

Attitude Items	Strongly Disagree *n* (%)	Disagree *n* (%)	Neither Agree nor Disagree *n* (%)	Agree *n* (%)	Strongly Agree *n* (%)	Median (IQR)
A1: Antimicrobial resistance is an important public health problem.	1 (0.3)	1 (0.3)	2 (0.5)	107 (28.8)	261 (70.2)	5 (1)
A2: The fact that a patient is taking an antibiotic increases the risk of developing resistance	5 (1.3)	26 (7.0)	38 (10.2)	200 (53.8)	103 (27.7)	4 (1)
A3: New antimicrobials development can solve antimicrobial resistance problem.	37 (9.9)	96 (25.8)	83 (22.3)	111 (29.8)	45 (12.1)	3 (2)
A4: The use of antimicrobials in livestock animals is an important cause of appearance of new resistance to pathogenic agents in humans.	5 (1.3)	20 (5.4)	68 (18.3)	177 (47.6)	102 (27.4)	4 (2)
A5: In all cases where antimicrobials are dispensed, it is essential that patients be advised about complying with the treatment	1 (0.3)	3 (0.8)	12 (3.2)	96 (25.8)	260 (69.9)	5 (1)
A6: Antimicrobials are sometimes dispensed without medical prescription because the patient is known to have difficulty in obtaining a medical consultation.	12 (3.2)	40 (10.8)	91 (24.5)	179 (48.1)	50 (13.4)	4 (1)
A7: Antimicrobials are sometimes prescribed without medical prescription because the patient is known to have neither the time nor the money to see a physician.	14 (3.8)	51 (13.7)	97 (26.1)	171 (46.0)	39 (10.5)	4 (1)
A8: Dispensing antimicrobials without prescription is serious issue.	31 (8.3)	85 (22.8)	88 (23.7)	113 (30.4)	55 (14.8)	4 (1)

**Table 4:** Community pharmacists’ practices relating to antimicrobials’ use and resistance.

Practice Items	Never *n* (%)	Occasionally *n* (%)	Fairly Often *n* (%)	Usually *n* (%)	Always *n* (%)	Median (IQR)
P1: I educate patients on the use of antimicrobials and resistance-related issues.	-	14 (3.8)	51 (13.7)	153 (41.1)	154 (41.4)	4 (1)
P2: I take part in antimicrobial awareness campaigns to promote the optimal use of antimicrobials	2 (0.5)	38 (10.2)	102 (27.4)	147 (39.5)	83 (22.3)	4(1)
P3: I lack continuing education in antimicrobial use and resistance topics.	79 (21.2)	196 (52.7)	75 (20.2)	18 (4.8)	4 (1.1)	2(1)
P4: I make efforts to prevent or reduce the transmission of infections within the community.	2 (0.5)	20 (5.4)	90 (24.2)	184 (49.5)	76 (20.4)	4(1)
P5: I collaborate with other health professionals for infection control and antimicrobial stewardship	81 (21.8)	104 (28.0)	67 (18.0)	85 (22.8)	35 (9.4)	3 (2)
P6: I ask the patient’s history and symptoms of their infections before deciding to dispense antimicrobials.	-	3 (0.8)	7 (1.9)	111 (29.8)	251 (67.5)	5 (1)
P7: I seek additional clinical information (e.g. drug interactions, ADRs, allergy, etc.) before deciding to dispense the antimicrobials	1 (0.3)	56 (15.1)	59 (15.9)	157 (42.2)	99 (26.6)	4 (2)
P8: I screen the antimicrobials in accordance with local guidelines before dispensing	-	5 (1.3)	57 (15.3)	186 (50.0)	124 (33.3)	4(1)
P9: I dispense antimicrobial with complete clinical information (e.g. drug interactions, ADRs, allergy, etc.)	1 (0.3)	20 (5.4)	75 (20.2)	176 (47.3)	100 (26.9)	4 (2)
P10: I dispense antimicrobials without a prescription.	1 (0.3)	18 (4.8)	69 (18.5)	124 (33.3)	160 (43.0)	4(1)

**Table 5:** Correlation of Knowledge, Attitude, and Practice scores.

	Spearman’s Rho	*p*-Value
**Knowledge–Attitude**	0.100	0.053
**Attitude–Practice**	0.149	0.004 ***
**Knowledge–Practice**	0.071	0.174

*** = significant at *p* value < 0.01 level.

**Table 6:** The relationship of KAP scores with the community pharmacists’ demographics.

Demographics	Knowledge Score			Attitude Score			Practice Score		
	Median (IQR)	Mean Rank	*p*-Value	Median (IQR)	Mean Rank	*p*-Value	Median (IQR)	Mean Rank	*p*-Value
**Gender** ^a^									
Male (*n* = 112)	7 (2)	197.49	0.183	29 (5)	185.5	0.905	38 (7)	204.97	0.029 **
Female (*n* = 260)	6 (1)	181.77		28 (5)	186.93		36 (6)	173.54	
**Age** ^b^									
lower than 30 yrs. (*n* = 145)	7 (2)	196.18	0.358	29 (4)	196.18	0.350	36 (6)	189.59	0.430
30–40 yrs. (*n* = 185)	6 (1)	179.96		28 (5)	178.63		37 (6)	188.66	
over than 40 yrs. (*n* = 42)	7 (1)	181.9		29 (6)	199.12		36 (8)	166.29	
**Degree** ^a^									
B Pharm (*n* = 287)	6 (1)	180.96	0.060 *	28 (4)	177.77	0.004 *	37 (7)	180.86	0.062 *
PharmD (*n* = 85)	7 (2)	205.22		30 (2)	215.99		37 (6)	205.55	
**Postgrad** ^a^									
None (*n* = 276)	7 (1)	190.06	0.265	29 (5)	187.12	0.849	37 (6)	188.41	0.560
PG degree (*n* = 96)	6 (1)	176.26		28 (5)	184.71		36 (7)	181.01	
**Experience** ^b^									
less than 5 yrs. (*n* = 180)	6.5 (2)	183.24	0.840	28 (4)	182.9	0.019 *	37 (7)	198.93	0.091 *
5–10 yrs. (*n* = 152)	6 (1)	189.92		28 (5)	178.96		36 (6)	178.96	
more than 10 yrs. (*n* = 40)	6.5 (1)	188.24		31 (5)	231.34		36 (7)	231.34	
**Location** ^a^									
BKK (*n* = 258)	7 (1)	189.1	0.470	28 (5)	188.84	0.528	37 (6)	189.04	0.492
CHON (*n* = 114)	6 (1)	180.61		29 (5)	181.21		37 (7)	180.75	
**Type** ^a^									
Chain pharmacy (*n* = 139)	7 (2)	199.18	0.071 *	29 (4)	206.16	0.006 *	36 (5)	180.36	0.394
Independent pharmacy (*n* = 233)	6 (1)	178.94		28 (5)	174.77		37 (7)	190.16	

a = Mann–Whitney U test, b = Kruskal–Wallis test, * = significant at *p*-value < 0.1, ** = significant at *p*-value < 0.05; BKK: Bangkok; CHON: Chonburi.

## Data Availability

Data is contained within the article.
